# I know better! Emerging metacognition allows adolescents to ignore false advice

**DOI:** 10.1111/desc.13101

**Published:** 2021-03-08

**Authors:** Madeleine E. Moses‐Payne, Johanna Habicht, Aislinn Bowler, Nikolaus Steinbeis, Tobias U. Hauser

**Affiliations:** ^1^ UCL Institute of Cognitive Neuroscience University College London London UK; ^2^ Max Planck UCL Centre for Computational Psychiatry and Ageing Research London UK; ^3^ Wellcome Centre for Human Neuroimaging University College London London UK; ^4^ Division of Psychology and Language Sciences University College London London UK

**Keywords:** adolescence, advice, decision making, development, introspection, metacognition

## Abstract

Adolescents aspire for independence. Successful independence means knowing when to rely on one's own knowledge and when to listen to others. A critical prerequisite thus is a well‐developed metacognitive ability to accurately assess the quality of one's own knowledge. Little is known about whether the strive to become an independent decision maker in adolescence is underpinned by the necessary metacognitive skills. Here, we demonstrate that metacognition matures from childhood to adolescence (*N* = 107) and that this process coincides with greater independent decision‐making. We show that adolescents, in contrast to children, take on others’ advice less often, but only when the advice is misleading. Finally, we demonstrate that adolescents’ reduced reliance on others’ advice is explained by their increased metacognitive skills, suggesting that a developing ability to introspect may support independent decision‐making in adolescence.

## Graphical abstract

Moses‐Payne et al investigated how advice taking develops during childhood and adolescence, and how this is related to metacognition. Children, 8‐9 year olds (yo), and two groups of adolescents, 12‐13 year olds and 16‐17 year olds, took part in a metacognition and advice taking task. Both adolescent groups showed increased ability to identify when they were correct or incorrect (increased metacognitive efficiency, Panel A). The adolescent groups also showed a lower propensity to follow advice than children (Panel B). Importantly, adolescents were less likely to follow false advice but still followed helpful advice (Panel C). In this way, adolescents were more competent advice takers, and this was linked to their metacognitive abilities: resistance to false advice was driven by adolescents' increased metacognitive efficiency (Panel D). The adolescents utilized their new‐found metacognitive abilities to resist false advice from others when they knew they were correct, but still took helpful advice when they knew they were incorrect.

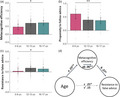

**Research Highlights**

- Metacognition matures from childhood to adolescence.
- Adolescents, in contrast to children, take on others’ advice less often, but only when the advice is misleading.
- Adolescents’ reduced reliance on others’ advice is explained by emerging metacognitive skills.

## 1 INTRODUCTION

Adolescents strive for independence, and are often accused of not listening to their parents’ advice. According to Socrates, “they have bad manners, contempt for authority; […] they contradict their parents […] and tyrannize their teachers” (Patty & Johnson, 1953). Today's stereotypes about teenagers often echo this sentiment that teenagers are indignant towards the wishes of their parents or teachers (Stern, 2005). Known as the ‘separation‐individuation’ arbitration (Koepke & Denissen, 2012), adolescence is a time when becoming independent from one's parents is particularly pertinent, as adolescents heed increasing control over their own decisions.

Becoming an independent decision maker is an important step towards full adult autonomy. However, resistance to authority (Kuhn & Laird, 2011) and increased risk‐taking behaviours (Duell et al., 2018) during this period have led to the assumption that adolescents still lack the necessary abilities for making good independent decisions. This stands, at least in part, in contrast to legal and constitutional rights that allow adolescents to make independent decisions without consulting others first (e.g. medical treatment decisions, online privacy consenting, driving, voting). What are the cognitive processes that enable us to become independent and good decision makers, and when do they develop?

A critical part of being an independent decision maker is the ability to accurately judge one's previous decisions (Batha & Carroll, 2007; Yeung & Summerfield, 2012). This metacognitive ability—the ability to accurately introspect on one's own decisions (Koriat, 2007)—is critical when deciding how much to rely on one's own decisions or on the advice of others (Fiedler et al., 2019). One common procedure for assessing metacognition is to ask participants for confidence judgements about their own performance in the absence of feedback. Previous work has indicated that children as young as 3 years old have some metacognitive ability, in that they are more confident when correct than when incorrect (Lyons & Ghetti, 2011). This distinction may continue to improve during childhood and into early adolescence, as children's confidence ratings become better aligned to their actual performance (Fandakova et al., 2017; Hembacher & Ghetti, 2013; Roebers et al., 2004). Fewer studies have assessed the continued development of metacognitive ability across adolescence (Brackmann et al., 2019; Weil et al., 2013).

In addition, independent decisions require the ability to utilise confidence signals to monitor behaviour, for example deciding when to seek further information or help from others and when to incorporate versus ignore such information. Previous work has shown that young children and even infants may be able to utilise their confidence in their own and others’ decisions to know when to ask for help (Coughlin et al., 2015; Goupil et al., 2016; Koenig & Harris, 2005; Lyons & Ghetti, 2011). Moreover, young children appear to be able to monitor the accuracy of advisors and choose the reliable among multiple advisors (Koenig & Harris, 2005). However, they may be misled when given false advice that contradicts their *own* decision or knowledge and, in such a scenario, often only ignore testimony from others when they can be highly confident in their own beliefs (Jaswal, 2010; Jaswal et al., 2010). As children approach adolescence, they become better able to arbitrate between recommendations from others and their own beliefs (Selmeczy & Ghetti, 2019), but may still be misled when information is not helpful (Roebers, 2002; Roebers & Howie, 2003; Schwarz & Roebers, 2006).

Evidence in probabilistic learning contexts suggests that adolescents perform similarly to (or sometimes better than; Decker et al., 2015) adults when they need to ignore false advice from others (Lourenco et al., 2015). In such paradigms, a participant is typically given a single instance of helpful or misleading advice at the beginning of the task before learning for themselves the value of a set of options (Decker et al., 2015; Lourenco et al., 2015; Rodriguez Buritica et al., 2019). This design may better capture the influence of increased exploration in adolescence, rather than advice taking behaviour *per se*, as participants can quickly determine the (in)validity of the advice by exploring other non‐advised options (Dubois et al., 2020; Rodriguez Buritica et al., 2019).

In the current study, we exploited recent methodological advances in the measurement of metacognition (Fleming & Lau, 2014; Maniscalco & Lau, 2012, 2014), so as to distinguish between participants’ overall confidence (metacognitive bias) and ability to track performance (metacognitive sensitivity). Metacognitive bias and sensitivity have often been conflated previously, but may track different psychological phenomena (Hauser et al., 2017; Moses‐Payne et al., 2019; Rollwage et al., 2018; Rouault, Seow, et al., 2018). We use a signal detection theory framework in the context of a simple perceptual discrimination task, which helps us to better tease apart these constructs and assess their relative contribution to development (Fleming & Lau, 2014; Maniscalco & Lau, 2012, 2014). Such an approach has been used previously with infants, adolescents and adults (Fandakova et al., 2017; Goupil & Kouider, 2016; Goupil et al., 2016; Salles et al., 2016; Weil et al., 2013), but still little is known about development from childhood to adolescence and how metacognitive bias and sensitivity relate to advice taking.

We designed a novel paradigm which controls for, potentially confounding, individual differences in perceptual performance by titrating performance using a staircase procedure (Cornsweet, 1962; Fleming et al., 2010; García‐Pérez, 1998; Levitt, 1971). Furthermore, we use metacognitive ‘efficiency’ (Fleming & Lau, 2014; Maniscalco & Lau, 2012, 2014) as a measure of how well participants’ confidence tracked their performance, which also accounts for any differences in the level of true performance (often overlooked in previous research). This metacognition task design was paired with advice from a ‘space advisor’ and helpful advice was contrasted with misleading advice at trial level. Advice was given after a decision had already been reached in order to assess changes of mind, which may be more tightly linked to metacognitive evaluations (Frith, 2012; Hauser et al., 2017; Koriat, 2012; Moran et al., 2015; Moses‐Payne et al., 2019; Rollwage et al., 2018; Yeung & Summerfield, 2012).

By combining metacognition and advice taking in one task, we were able to investigate how young people can utilise their emerging metacognitive abilities to arbitrate helpful from misleading advice. We assessed the interaction between confidence and advice taking in children (8–9 years), early adolescents (12–13 years) and late adolescents (16–17 years) to investigate protracted development of metacognitive ability and advice taking into the teenage years. We show that the emergence of metacognitive abilities during early adolescence drives independent decision‐making, in that it allows adolescents to ignore misleading advice but take on helpful advice from others.

## 2 METHODS

### 2.1 Participants

We recruited participants from multiple schools across London, UK. For participants under the age of 16, we gained parental and participant consent. Participants over the age of 16 consented to take part themselves. Participants were given a voucher valued at £7.00 for their participation and no aspects of the tasks were explicitly incentivised with monetary gain, consistent with previous work (e.g. Fandakova et al., 2017; Weil et al., 2013). The study was approved by the Research Ethics Committee of University College London (study number: 14261/001).

We tested 108 participants and analysed the data from 107 participants. One participant was excluded (due to a pre‐existing neurological condition). Out of 107 participants, 45 were male and 62 were female. We recruited participants in three age groups: age 8–9 (i.e. 8.89–9.71, mean age ± SD: 9.34 ± 0.27, *n* = 30, male/female *n* = 11/19), age 12–13 (i.e. 12.69–13.70, mean age ± SD: 13.13 ± 0.30, *n* = 41, m/f *n* = 19/22) and age 16–17 (i.e. 16.71–17.76, mean age ± SD: 17.19 ± 0.29, *n* = 36, m/f *n* = 15/21). This age range was selected to span pre‐, early‐ and mid‐adolescence. The age groups did not differ in IQ (see Table 1). Our power calculations based on previous related studies (Decker et al., 2015; Hauser et al., 2017; Lourenco et al., 2015; Selmeczy & Ghetti, 2019; Weil et al., 2013) suggested a sample size of ~30 per group would be sufficient to detect medium size effects with 80% power. We optimised power analysis to detect age differences in our main dependent variables (metacognitive bias and sensitivity, advice taking), as this was our primary interest. We did not conduct a power analysis for our mediation analysis, as this was a secondary analysis. Previous studies have shown that mediation effects can be detected in a similar design with smaller samples than was recruited in the current study (Rollwage et al., 2020), but other studies suggest that substantially larger sample sizes are required (Fritz & MacKinnon, 2007; Schoemann et al., 2017).

**TABLE 1:** Participant demographics. Age, sex and IQ scores for the three age groups: 8–9 year olds, 12–13 year olds and 16–17 year olds. Statistical tests of the difference between groups are reported where applicable

	8–9 year olds (*n* = 30)	12–13 year olds (*n* = 41)	16–17 year olds (*n* = 36)	
Age, mean ± SD	9.34 ± 0.27	13.13 ± 0.30	17.19 ± 0.29	NA
Sex, m/f	11/19	19/22	15/21	*χ* ^2^(2) = 0.67, *p* = 0.716.
IQ (WASI‐II), mean ± SD	93.87 ± 13.44	98.51 ± 13.45	97.22 ± 10.26	*F*(2,104) = 1.24, *p* = 0.294

Abbreviation: WASI‐II, Wechsler Abbreviated Scale of Intelligence, version two.

To counteract the currently dominant recruitment bias towards higher socioeconomic status young people (Fakkel et al., 2020), we deliberately selected schools in socially diverse and disadvantaged areas. In participating schools, the proportion of pupils eligible for pupil premium (additional funding for children in local authority care or those known to be eligible for free school meals), with English as an additional language and from minority ethnic backgrounds was above or well above the national average according to Ofsted reports (Office for Standards in Education, 2013‐15).

### 2.2 Overview of procedure

Participants were introduced to the Space Explorer task and given both verbal and written instructions. Participants also completed three other tasks, some questionnaires (tasks and questionnaires reported elsewhere: Bowler et al., 2021; Dubois et al., 2020), and the Wechsler Abbreviated Scale of Intelligence verbal and abstract reasoning tests, version two (Wechsler, 2011; WASI‐II). Participants were tested in groups of 3–4 in a quiet room with two experimenters present. The order in which participants completed the tasks, questionnaires and WASI‐II was pseudo‐randomised between participants. In total, the adolescent participants (12–13 years, 16–17 years) spent ~1.5 h completing the experiment. The youngest participants (8–9 years) completed the experiment over two sessions to reduce fatigue (however, the Space Explorer task was always completed within a single session), and spent ~2 h completing the experiment.

### 2.3 Experimental design

#### 2.3.1 Space explorer task

##### Stimuli

**FIGURE 1:**
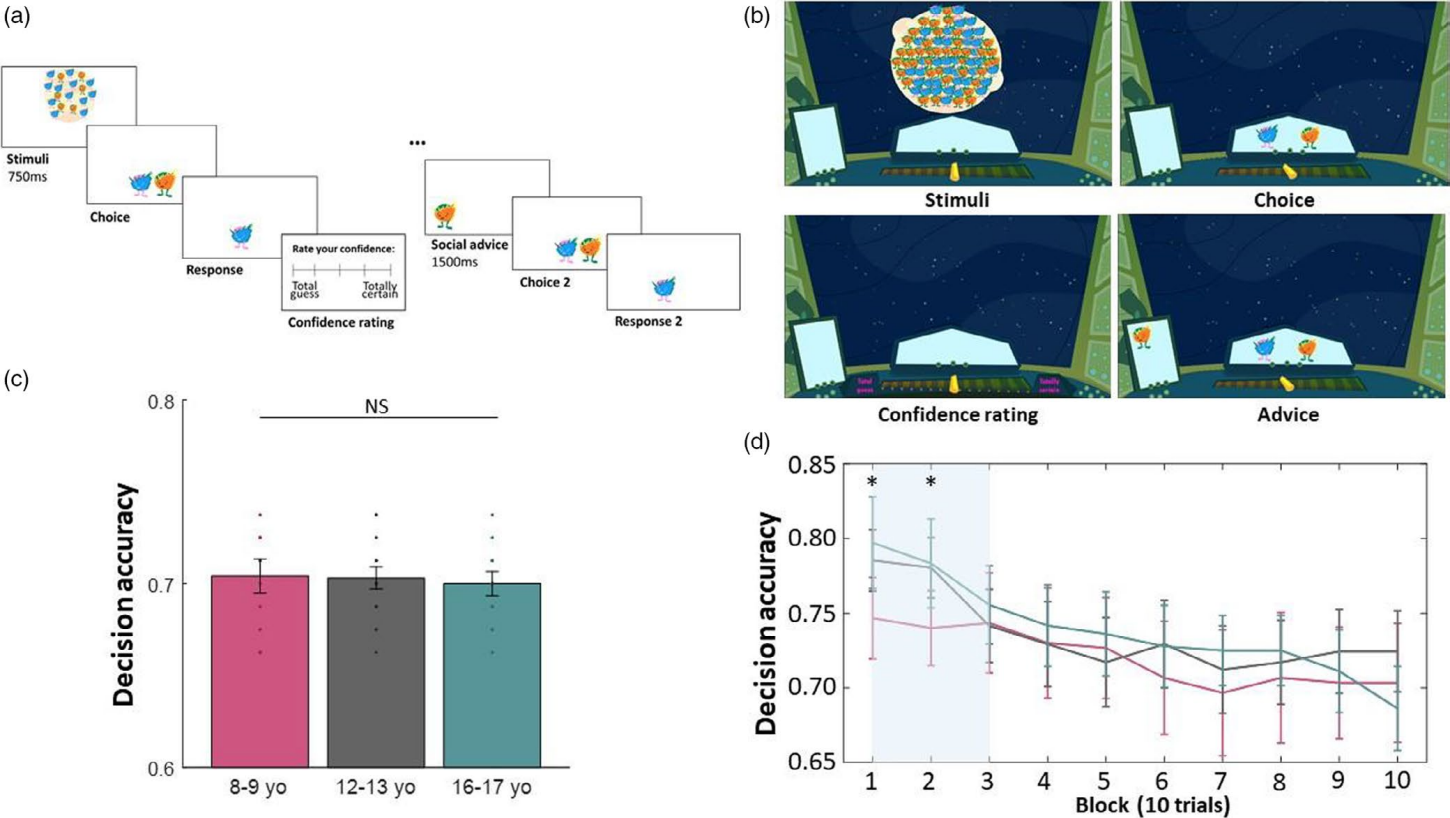
Probing advice taking in metacognition. We developed a novel task that allowed us to assess the development of metacognition and advice taking. (a) Participants viewed an array of two different coloured aliens for 750 ms, were asked to indicate which colour was more plentiful and rated their confidence in their decision. Subsequently, participants received advice from a ‘space advisor’, and had the opportunity to revise their choice. All decisions and confidence ratings were self‐paced. (b) The participants viewed a spaceship cockpit with two screens that displayed instructions, choices (middle screen) and advice (left screen) through the task. The planets were displayed through the cockpit ‘window’. (c) Task performance was staircased to achieve equal performance for all participants. Mean decision accuracy did not differ between age groups and was well calibrated to ~70% accuracy. Mean accuracy was very similar (NS, non‐significant) across age groups (8–9 years old [yo] M = 70.42; 12–13 yo M = 70.30; 16–17 yo M = 70.00). (d) Decision accuracy by blocks of 10 trials (colours represent age groups as in c). Shaded area indicates initial practice trials without confidence ratings that were excluded from any analysis. Error bars represent 95% confidence intervals. *p < 0.05 uncorrected

The Space Explorer task was programmed in Cogent 2000 (MATLAB toolbox, http://www.vislab.ucl.ac.uk/cogent_2000.php). Participants viewed a spaceship cockpit, within which were two display screens (one central for displaying choice, one to the side for displaying advice) and a confidence slider (displayed only during confidence reports; Figure 1b).

Stimuli for the perceptual decision consisted of a planet, presented briefly in the centre of the screen with 68 aliens displayed in a circle formation over the top of the planet. There were eight possible alien colours (selected to be easily discriminated between, even by participants with colour blindness) but on each trial two different colour aliens were randomly selected. Aliens were identical apart from differences in colour. There was always more of one colour alien than the other, the exact difference in number was calibrated to individual participants based on a staircase procedure to ensure equal performance (see below). After stimulus presentation, an example of the two aliens was displayed on the left and right of the central display screen for participants to make their choice.

##### Task procedure

In the Space Explorer task, participants were first asked to make a perceptual decision based on an array of two different colour aliens presented for 750 ms. Participants were instructed to decide as quickly as possible (‘as quickly as you can’), which of the two aliens there was more of and log their response by key press.

This was followed by a confidence rating. Participants rated their confidence on a sliding scale from ‘total guess’ to ‘totally certain’, labels that were chosen after piloting to ensure the youngest participants would comprehend the scale. Participants were instructed to use the entire length of the scale.

In half of the trials, this was then followed by advice from a space advisor. The advised answer (an image of one alien) was displayed to participants for 1500 ms in a small ‘messenger screen’. Participants were then given the option to revise their initial choice and rerated their confidence (see Supporting Information for analysis of second confidence rating). The advice was correct on 70% of trials, thus matching the participants’ performance. This level of accuracy of advice was chosen in order to avoid stereotyped responses from the participant (e.g. advice that is totally accurate may lead participants to always follow advice and ignore their own choices, whereas advice that is chance‐level may lead to participants completely ignoring advice). The trials on which advice was given and on which the advice was correct or incorrect (70% correct trials) were randomly determined. Participants were instructed that (in verbal instructions) ‘the advisor should be correct most of the time but can also make mistakes’ (and reiterated in the written instructions, given by a masked astronaut) ‘the advisor should be correct most of the time, but they're only human, so can make mistakes just like you and I!’, but were not given any indication of any other characteristics of the advisor.

For the practice trials (first 30 trials), participants simply made the perceptual decision and were given feedback (‘correct’ vs. ‘incorrect’). We did not find any evidence that the amount of positive versus negative feedback in the practice trials had an influence on confidence‐related measures in the main task (see Supporting Information). These trials were included to allow the staircase to settle before starting the task (Figure 1d). Feedback was given on practice trials in line with previous work (e.g. Moses‐Payne et al., 2019; Rollwage et al., 2018), to allow faster convergence and so that experimenters could monitor performance to ensure participants understood the task. In the remaining trials (80 trials), participants were no longer given feedback but instead rated their confidence in their response. For half of these trials, participants received advice, revised their initial choice and rerated their confidence.

All pre‐ and post‐advice decisions and confidence ratings were self‐paced. In total, the Space Explorer task took 10–20 min to complete. For task procedure see Figure 1a, for task interface see Figure 1b.

##### Calibration

A staircase procedure was used throughout the task in order to match participants’ performance. To this end, we used a 2‐down‐1‐up staircase procedure that converged at ~70% accuracy (Cornsweet, 1962; Fleming et al., 2010; García‐Pérez, 1998; Levitt, 1971; see Supporting Information). This was used to identify the difference in aliens needed to elicit near‐threshold performance (i.e. between chance and ceiling performance) for individual participants so as to elicit the most variation in confidence ratings (as previously, e.g. Fleming et al., 2010). The staircase could also account for any differences in speed‐accuracy trade‐offs between groups by adjusting evidence strength accordingly.

### 2.4 Statistical analysis

#### 2.4.1 Model comparison approach to analysis

The development of cognitive functions from childhood to adolescence does not always follow a simple linear trajectory with age (Jones et al., 2014; Nook et al., 2019; Somerville et al., 2013, 2017; Van Den Bos et al., 2015). For this reason, we used a model comparison approach to compare between linear, quadratic and adolescent‐emergent (appears in early adolescence and then plateaus) patterns of developmental differences. This approach has been used previously to better describe nonlinear patterns of development across this age range (e.g. Jones et al., 2014; Nook et al., 2019; Somerville et al., 2013, 2017; Van Den Bos et al., 2015). Linear age was computed using *z*‐scored raw age; quadratic age was computed using the square of linear age; adolescent‐emergent age was calculated using quadratic age but replacing values above 12 with the same value (Somerville et al., 2013; see Figure S4 for age curves). For each analysis, we compared the model fit of these three models of age and the linear combinations (sums) thereof (seven models in total) using Bayesian Information Criterion (BIC) to determine the best fitting model (BIC tables and full results reported in Supporting Information; see Table S1).

We report the winning model for each analysis using continuous transformed age and we use subsequent independent *t*‐tests to compare between age groups. We plot effects using age groups for interpretability. We also include sex as a covariate in all models, as previous research has suggested sex may be associated with confidence‐based measures in adolescence (Weil et al., 2013). Although we report significant effects of sex where they were found, sex was not a main predictor variable of interest and we did not make any a priori hypotheses about sex. To compare performance across groups, we use ANOVA. We report effect sizes using Cohen's d and partial eta‐squared where applicable.

#### 2.4.2 Metacognitive bias and sensitivity

We calculated metacognitive bias (named in line with previous literature e.g. Fleming & Lau, 2014; Moses‐Payne et al., 2019; Rouault, Seow, et al., 2018) by taking the mean confidence rating across all trials in which confidence ratings were given. Given that basic task performance was equated between subjects, mean confidence ratings reveal between‐subject ‘bias’ in subjective confidence (rating generally high or low confidence).

We calculated metacognitive efficiency (meta‐d′/d′) using signal detection theory approach and maximum likelihood estimation using Matlab (Maniscalco & Lau, 2012, 2014; function provided by http://www.columbia.edu/~bsm2105/type2sdt/). Meta‐d′ quantifies type 2 metacognitive sensitivity (the degree to which participants’ confidence ratings discriminate between correct and incorrect trials) and is expressed in the same units as type 1 perceptual sensitivity or d′ (the degree to which participants can distinguish between different coloured aliens). Metacognitive efficiency (meta‐d′/d′) is a relative measure that eliminates any remaining performance and response bias confounds (Barrett et al., 2013; Galvin et al., 2003; Maniscalco & Lau, 2012, 2014; Masson & Rotello, 2009). Perfect metacognition occurs when meta‐d′ and d′ are matched and thus meta‐d′/d′ is 1. For further detail see Supporting Information.

Meta‐d′ is theoretically bounded at the lower end by zero, but when fit using an unbounded maximum likelihood procedure estimation error may lead to negative values in practice. This estimation error, of course, applies to all values but becomes evident when values fall outside the theoretical range (Fleming et al., 2014; Maniscalco & Lau, 2012). On these grounds, we did not see sufficient reason to remove or adjust negative values and report analyses including negative values. For completeness, however, we did repeat analyses in which we set negative meta‐d′ values to zero and report the same pattern of results (see Supporting Information; Figure S7). Three participants (aged 8–9) gave mostly extreme confidence ratings and thus their metacognitive sensitivity (and subsequently metacognitive efficiency) could not be estimated, these participants were thus not included in subsequent analyses that included metacognitive efficiency. We also performed all analyses using metacognitive sensitivity (meta‐d′) rather than metacognitive efficiency and found the same pattern of results (data not shown).

#### 2.4.3 Advice taking and resistance to false advice

Overall propensity to follow advice was calculated by taking the proportion of trials that participants switched their choice when the advisor disagreed with them (over total number of trials where advisor disagreed) minus the proportion of trials that participants switched their choice when the advisor agreed with them (over total number of trials where the advisor agreed). This was done to account for changes of mind that were not advice related.

Resistance to false advice was calculated by taking the proportion of trials in which participants followed helpful advice, that is were incorrect and switched to the advised correct choice (over total number of trials where participants were incorrect and received conflicting advice) minus the proportion of trials in which participants followed misleading advice, that is were correct and switched to the advised incorrect choice (over the total number of trials where participants were correct and received conflicting advice). Therefore, a score of zero means that the participant did not discriminate between helpful and misleading advice in their second choice, a positive score means the participant followed helpful more than misleading advice and a negative score means the participant followed misleading more than helpful advice.

Both propensity to take advice and resistance to false advice were standardised (*z*‐scored).

#### 2.4.4 Mediation analysis

We used mediation analysis as a secondary analysis to assess whether the effect of age on resistance to false advice was mediated by metacognitive efficiency. We use standard notation to report mediation paths, where X represents the independent variable (adolescent‐emergent age), Y represents the outcome variable (resistance to false advice) and M represents the mediating variable (metacognitive efficiency, meta‐d′/d′). The *c* path defines the overall effect of X on Y or the total effect; *c’* defines the effect of X on Y controlling for M and represents the direct effect; *b* represents the effect of M on Y, controlling for X; *a* defines the effect of X on M and the product *ab* defines the indirect effect of X on Y through M.

We used Mediation Toolbox in Matlab (https://github.com/canlab/MediationToolbox; Wager et al., 2008, 2009) to perform the analysis. This toolbox is used to calculate mediation analysis based on a standard three‐variable path model (Baron & Kenny, 1986) with a bootstrap test for the statistical significance of the product *ab* (adjusted indirect effect of age on resistance to false advice; Efron & Tibshirani, 1986; Shrout & Bolger, 2002). We entered adolescent‐emergent age as the predictor variable, resistance to false advice (standardised) as the outcome variable, metacognitive efficiency (meta‐d′/d′, standardised) as the mediator and sex was entered as the covariate (as in all previous analyses). This toolbox tests the significance of *ab* using the accelerated, bias‐corrected bootstrap test (Efron & Tibshirani, 1986; Shrout & Bolger, 2002) with 10,000 bootstrap samples to test each of the *a*, *b* and *ab* path coefficients. We required that all three paths (*a*, *b* and *ab*) were significant in order to satisfy the conclusion that the covariance between adolescent‐emergent age and resistance to false advice was explained by metacognitive efficiency.

## 3 RESULTS

To test the hypothesis that advice taking during adolescent development is linked to an improved metacognitive efficiency, we tested three age groups of participants, 8–9 years, 12–13 years and 16–17 years (selected to span pre‐, early‐ and late‐adolescence). Participants completed the Space Explorer task, in which they made perceptual decisions, rated their confidence in those decisions and, on some trials, were able to revise initial decisions after receiving advice.

### 3.1 Perceptual decision‐making performance was matched across age groups

To assess participants’ metacognitive abilities without bias due to differences in decision‐making performance, we used a staircase procedure in our task that enabled participants in each age group to achieve the same level of accuracy in their perceptual judgements. The staircase procedure was successful, as participants did not differ in their overall task performance (Figure 1c; *F*(2,104) = 0.36, $\eta_{p}^{2}$ = 0.01, *p* = 0.700), nor at any point during the task (Figure 1d). Further details on the groups’ perceptual performance, the staircase procedure and evidence differences required to achieve matched performance are reported in Supporting Information (Figures S1–S3).

Age groups did not differ in IQ (as measured by WASI‐II, *F*(2,104) = 1.24, *p* = 0.294; see Table 1) and IQ did not relate to any task‐based measures (decision accuracy: *β* = 0.0008, *SE* = 0.002, *p* = 0.685; metacognitive efficiency: *β* = 0.05, *SE* = 0.05, *p* = 0.263; propensity to follow advice: *β* = 0.01, *SE* = 0.02, *p* = 0.627; resistance to false advice: *β* = −0.14, *SE* = 0.09, *p* = 0.117; all results remained when controlling for IQ).

### 3.2 Early adolescents and males were most confident

All age groups appeared to use the scale in a similar way, in that confidence ratings were well distributed and were similarly variable across age groups (standard deviation of confidence ratings: *F*(2,104) = 1.63, *p* = 0.202; see Figures S5 and S6).

**FIGURE 2:**
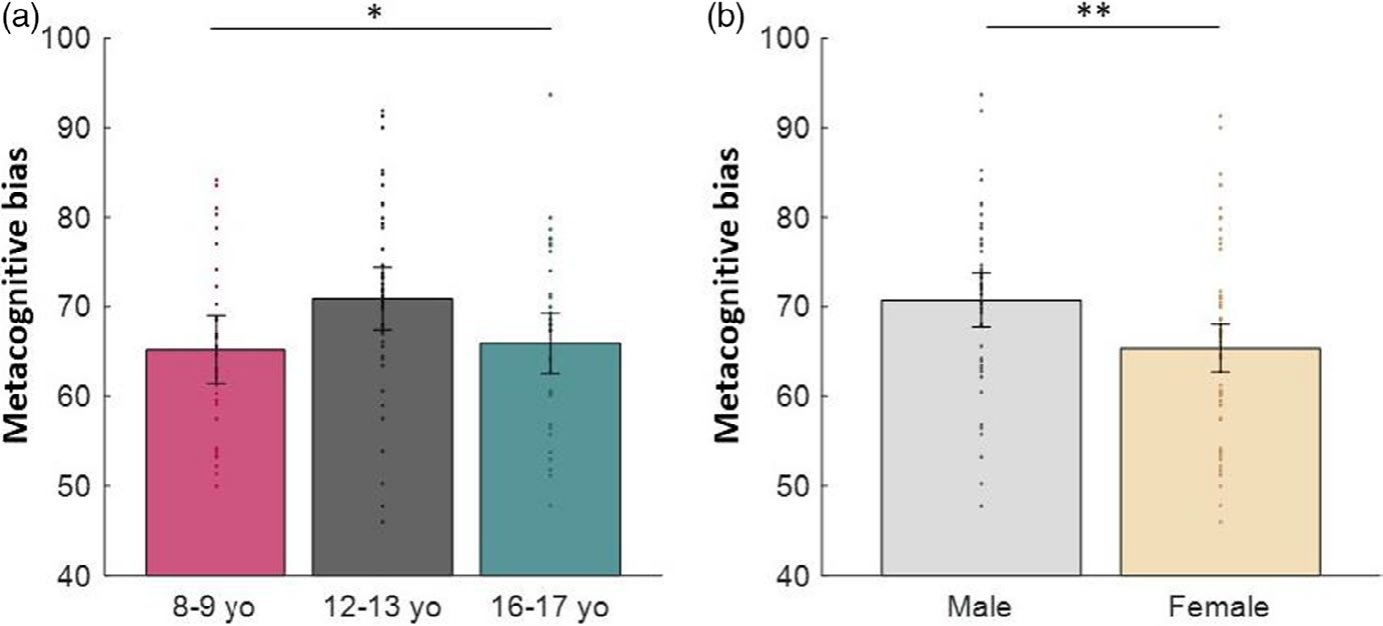
Altered mean confidence (metacognitive bias) in early adolescents and males. (a) 12–13 year olds show heightened mean confidence compared with other age groups. (b) Male participants report heightened confidence compared to females. Error bars represent 95% confidence intervals. *p < 0.05, **p < 0.01

To assess participants’ metacognitive bias, we examined the mean confidence score across all trials. Interestingly, we found that metacognitive bias peaked in the 12–13 years group, showing that this age group was more confident than the other groups (Figure 2a; continuous quadratic age: *β* = 0.26, *SE* = 0.12, *p* = 0.029, controlling for sex) despite performing at the same level on the perceptual judgements (Figure 1c). Subsequent comparisons show significant difference in mean confidence between 8–9 years and 12–13 years (*t*(69) = −2.23, *d* =0.54, *p* = 0.029) and between 12–13 years and 16–17 years (*t*(75) = 2.08, *d* = 0.48, *p* = 0.041) but not between 8–9 years and 16–17 years (*t*(64) = −0.28, *d* = 0.07, *p* = 0.779).

In addition, males were also more confident than females (Figure 2b; *β* = 0.25, *SE* = 0.09, *p* = 0.009, controlling for age) despite matched performance (*t*(105) = 0.30, *d* = 0.52, *p* = 0.764).

### 3.3 Adolescents have better metacognitive ability

Whilst metacognitive bias provides information about overall confidence, it does not provide any information about how well confidence ratings were calibrated to participants’ actual performance. Metacognitive efficiency, on the other hand, measures how well participants’ confidence ratings aligned with their performance.

**FIGURE 3:**
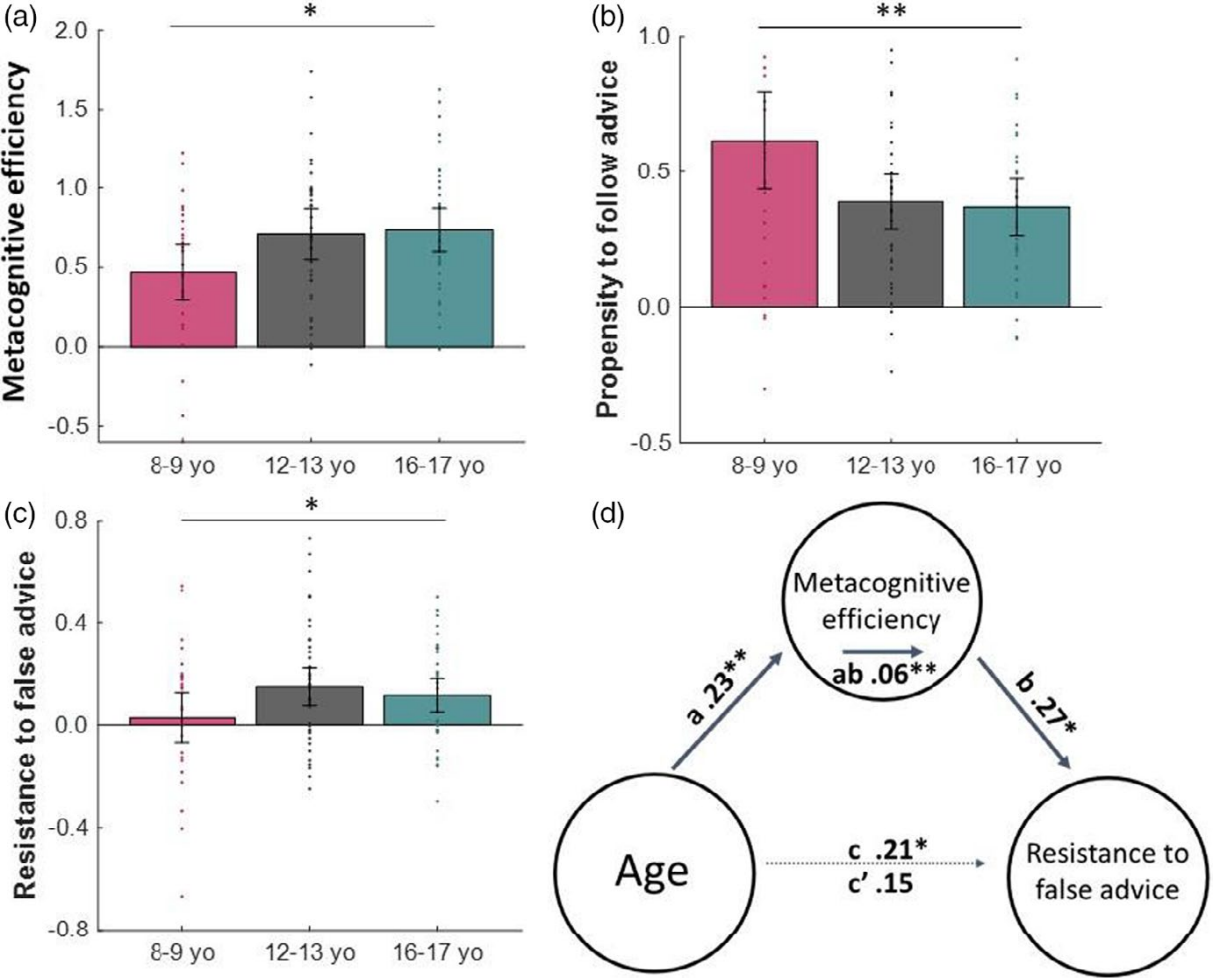
Metacognitive efficiency influences advice taking behaviour in adolescents. (A) Both adolescent groups show greater metacognitive efficiency (meta‐d′/d′), indicating that metacognition increased primarily between childhood and adolescence. (B) In addition, we found significant developmental changes in advice following. The youngest were the most likely to follow advice compared to adolescent groups (calculated as the proportion of trials that participants switched their choice when the advisor disagreed with them, over total number of trials where advisor disagreed, minus the proportion of trials that participants switched their choice when the advisor agreed with them, over total number of trials where the advisor agreed). (C) Both adolescent groups were better able to resist false advice while still taking on helpful advice (calculated as the proportion of trials that participants were incorrect and switched to the advised correct choice rather than sticking with their incorrect choice minus the proportion of trials that participants were correct and switched to the advised incorrect choice rather than sticking with their correct choice). Children, however, were not able to discriminate helpful from misleading advice. (D) The relationship between age (adolescent‐emergent) and resistance to false advice was mediated by metacognitive efficiency. Mean beta values are shown, the *c* path represents the total effect of age on resistance to false advice and *c′* represents the effect of age on resistance to false advice when controlling for the mediator metacognitive efficiency. All graphs show raw values (before *z*‐scoring). Error bars represent 95% confidence intervals. *p < 0.05, **p < 0.01

We found an improvement in metacognitive efficiency in an adolescent‐emergent pattern across age groups. Both adolescent groups (12–13 year olds and 16–17 year olds) had better metacognitive efficiency than the pre‐adolescent group (8–9 year olds; continuous adolescent‐emergent: *β* = 0.23, *SE* = 0.10, *p* = 0.023; Figure 3a). Subsequent comparisons showed the effect was primarily driven by different metacognitive efficiency between 8–9 years and 16–17 years (*t*(61) = −2.48, *d* = 0.63, *p* = 0.016), 12–13 years showed somewhat better metacognitive efficiency than 8–9 years (*t*(66) = −1.99, *d* = 0.50, *p* = 0.051) and the adolescent groups showed very similar metacognitive efficiency (12–13 years vs. 16–17 years: *t*(75) = −0.26, *d* = 0.06, *p* = 0.795). This means the confidence reports given by the adolescent groups were better calibrated to their actual performance than the confidence reports given by the pre‐adolescent group.

### 3.4 Adolescents are less willing to take (misleading) advice from others

Next, we assessed how children and adolescents incorporate advice from others into their perceptual judgements. Using a post‐decision paradigm (Meshi et al., 2012), we could assess how advice was weighted against participants’ own initial performance and how this was related to their confidence.

To assess participants’ overall propensity to follow advice, we first calculated the difference between the proportion of trials that participants changed their minds when the advisor disagreed versus when the advisor agreed with them (accounting for task‐irrelevant switching behaviour). We found an (inverse) adolescent‐emergent pattern, showing that the adolescent participants generally followed advice less than the youngest participants (Figure 3b; continuous adolescent‐emergent: *β* = −0.26, *SE* = 0.09, *p* = 0.005), supporting the idea that adolescents are generally resistant to others’ opinions. Subsequent comparisons showed propensity to follow advice was significantly higher in the 8–9 years compared with 12–13 years (*t*(69) = 2.04, *d* = 0.48, *p* = 0.044) and compared with 16–17 years (*t*(69) = 2.35, *d* = 0.57, *p* = 0.022) but propensity to follow advice was not different between the two adolescent groups (*t*(75) = 0.50, *d* = 0.12, *p* = 0.615).

Given that advice is reliable in this task, following advice overall is sensible. However, a good decision maker will also take into account their own performance, weighing up the advice against their own decisions. Does this mean that the youngest participants, in contrast, are simply following advice blindly? To investigate resistance to false advice, we looked only at trials on which the advice was conflicting and calculated a difference score between the proportion of trials when participants were incorrect and followed the helpful advice (out of total number of trials participants were incorrect and received conflicting advice) and the proportion of trials when participants were correct but followed the misleading advice (out of total number of trials where participants were correct and received misleading advice).

A resistance score of zero means the participant did not discriminate between helpful and misleading advice when making their revised choice, a positive score means the participant followed helpful more than misleading advice and a negative score means the participant followed misleading more than helpful advice.

We found that the adolescent groups, compared with the youngest group, were more resistant to false advice and more willing to follow helpful advice (Figure 3c; continuous adolescent‐emergent: *β* = 0.20, *SE* = 0.10, *p* = 0.037). This shows that adolescents took their own performance into account when deciding whether to follow advice or not and because of this were better able to ignore misleading advice. By contrast, children tended to follow conflicting advice equally when it was helpful and when it was misleading. Subsequent comparisons showed the effect was mainly driven by a difference in resisting false advice between 8–9 years and 12–13 years (*t*(69) = −2.03, *d* = 0.48, *p* = 0.046) rather than between the other groups (8–9 years vs. 16–17 years *t*(64) = −1.55, *d* = 0.38, *p* = 0.127; 12–13 years vs. 16–17 years *t*(75) = 0.68, *d* = 0.16, *p* = 0.496). This suggests that adolescents do not simply ignore advice from others, but instead carefully examine their own knowledge and follow advice when they distrust their own decision.

### 3.5 Better metacognitive ability promotes adolescents’ ability to ignore misleading advice

So far, we observed two parallel age‐related effects. Adolescents had better metacognition i.e. were better able to take account of their own performance when deciding whether to follow advice. In addition, we showed that adolescents were better at arbitrating between their own decision and others’ advice. We were thus interested in whether emerging metacognition was the driving factor behind improved advice taking. Simply speaking, the better our insight into our own performance, the more our confidence signals reflect our actual performance and the more we can utilise these confidence signals to resist misleading advice from others. To assess whether the adolescents’ increased metacognitive efficiency allowed them to resist false advice but still take on helpful advice, we conducted a secondary mediation analysis assessing whether metacognition mediated the observed association between adolescent‐emergent age and advice taking (see Section 2.4 for details).

Our mediation analysis confirmed the significant associations between adolescent‐emergent age and metacognitive efficiency (path *a*: mean *β* = 0.23, *SE* = 0.09, *z* = 2.74, *p* = 0.006) and between adolescent‐emergent age and resistance to false advice (path *c*: mean *β* = 0.21, *SE* = 0.10, *z* = 2.26, *p* = 0.024). Moreover, we found a significant association between metacognitive efficiency and resistance to false advice (path *b*: mean *β* = 0.27, *SE* = 0.11, *z* = 2.33, *p* = 0.020). We found that the association between adolescent‐emergent age and resistance to false advice was fully mediated by metacognitive efficiency (*ab*: mean *β* = 0.06, *SE* = 0.04, *z* = 2.61, *p* = 0.009), and the association between adolescent‐emergent age and resistance to false advice was no longer significant when accounting for metacognitive efficiency (*c’*: mean *β* = 0.15, *SE* = 0.09, *z* = 1.65, *p* = 0.098). See Figure 3d; More details on path notation can be found in ‘Section 2.4’.

This pattern of results shows that adolescents’ ability to more accurately identify when they were correct and when they were incorrect allowed them to ignore advice more often when it was misleading but incorporate advice when it was helpful. In contrast, children were less able to identify when they were correct or incorrect and so tended to follow conflicting advice independent of their own performance and whether the advice was helpful or misleading. This suggests that adolescents do not simply ignore advice from others but use their confidence signals to guide their advice taking behaviour.

## 4 DISCUSSION

To understand when successful independent decision‐making emerges, we studied the development of metacognition and advice taking. Our findings provide evidence that metacognitive efficiency matures from childhood to adolescence. Furthermore, we show that this process supports more sensible advice taking behaviour. In this way, adolescents were able to use their confidence signals successfully to reject misleading advice but take on helpful advice from others. Adolescents followed conflicting advice less often when they were correct, but took it when they were incorrect. This is because adolescents’ confidence signals better discriminated between correct and incorrect trials, so they were better able to use their confidence signals to determine when to follow advice. ‘I know better! And I know I know better!’.

The development of metacognition, that is gaining better insight into our own behaviour, in adolescence may be an important driver for independent decision‐making. Our results suggest metacognitive efficiency and the ability to ignore false advice improve in the transition from childhood to adolescence. The best‐fitting model described an adolescent‐emergent trajectory of development, with major improvement in metacognitive ability and advice taking across children and early adolescents that remained the same for late adolescents. This indicates that the transition from childhood into adolescence is a developmental period of substantial change in metacognitive and cognitive processes. This trajectory was well represented by our data (see Figure S4) even though we collected participant data in age groups rather than continuously sampling across childhood and adolescence (as previously, e.g. Somerville et al., 2013). Importantly, all our continuous analyses are supported by subsequent between‐groups tests showing the same effect.

This, to our knowledge, is the first investigation into the impact of metacognitive development across childhood and adolescence on advice taking behaviour. We extend previous work on post‐decision evidence integration, that is how individuals incorporate new information after a decision has been reached (Moran et al., 2015; Moreira et al., 2018; Rollwage et al., 2020; van den Berg et al., 2016; Yeung & Summerfield, 2012), to show that metacognitive efficiency at the initial decision underlies sensible integration of information post‐decision (i.e. advice). Our results show that when adolescents received conflicting advice, they changed their mind more when they were incorrect than when they were correct (i.e. they change their mind in a more sensible way than children).

This study aimed to address some of the methodological issues raised by previous research. We used a signal detection theoretic approach for the measurement of metacognition to be able to dissociate metacognitive bias from efficiency. Therefore, our finding that metacognitive efficiency improves across childhood into adolescence is resistant to any developmental differences in overall confidence. We employed a perceptual decision‐making paradigm with adaptive difficulty in order to fine‐tune participants’ level of accuracy. We also used metacognitive efficiency to measure how well participants discriminated correct from incorrect decisions while accounting for any remaining inter‐individual performance differences. The current study offers a different angle to previous work in the development of metamemory. Recent work comparing different forms of metacognition and metamemory has suggested that metacognitive ability for perception is only weakly or not at all related to metamemory (Mazancieux et al., 2020; Rouault, McWilliams, et al., 2018). Moreover, patients with lesions to anterior prefrontal cortex show domain‐specific impairment in perceptual but not memory‐related metacognitive ability (Fleming et al., 2014). This was not the case for metacognitive biases in perceptual versus metamemory confidence, which are more closely related and may be domain‐general (Mazancieux et al., 2020; Rouault, McWilliams, et al., 2018). This again highlights the importance of distinguishing these measures.

This research makes an important contribution to a wider literature on age of mental (decision‐making) capacity, the age at which a person is able to make autonomous decisions, for example, about their own welfare (e.g. deciding between medications) or whether to enter into contractual agreements (e.g. buying property). There are multiple criteria that determine an individual's decision‐making capacity (Department of Health, Department for constitutional Affairs, & Welsh Assembly Government, 2005). The current study may be particularly relevant for the criterion of ‘use or weigh’, the ability to utilise multiple sources of information and weigh up their importance as part of the process of making a decision (Case, 2016; Grootens‐Wiegers et al., 2017; Ruck Keene et al., 2019; van der Plas et al., 2019). Thus, a person's ability to weigh the advice of others against their own confidence in their decision is an important aspect of capacity‐related decision‐making (van der Plas et al., 2019). We have shown here that adolescents’ maturing metacognition contributes to their ability to weigh up advice from others in a more sensible way, not just following advice whenever someone disagrees. A further understanding of the mechanisms that drive advice taking behaviour and adolescent decision‐making will improve our understanding of the development of these important requirements for decision‐making capacity.

Interestingly, we additionally found that early adolescents (12–13 years) showed heightened confidence compared with children and late adolescents (quadratic trajectory), in spite of the same level of performance across all age groups and in both sexes. This diverges from previous work suggesting that children are overconfident in judgements‐of‐learning (e.g. van Loon et al., 2017; Was & Al‐Harthy, 2018). However, in studies with retrospective confidence judgements as in the current study, the results are mixed. Sometimes, children do not differ from early adolescents in their overall confidence judgements (7–9 year olds were not more confident overall than 9–12 year olds; Fandakova et al., 2017; no clear pattern of overconfidence with age across 7–16 years; Brackmann et al., 2019) or overall confidence appears to increase across adolescence (increase in overall confidence across 11–17 years; Weil et al., 2013). Relatively fewer studies have assessed retrospective trial‐by‐trial confidence in late childhood and adolescence and the existing studies cover different age groups, different experimental paradigms and different ways of calculating overall confidence. It would be interesting to synthesise results across different techniques to address these conflicting findings in further studies.

We also found that males were more confident than females. A previous study on metacognition during adolescence found sex‐related differences in metacognition (Weil et al., 2013), but this was in metacognitive ability (measured by A_roc_) rather than mean confidence (as reported here). Sex differences in metacognition are not reliably reported in young children (Hembacher & Ghetti, 2013; Jaswal, 2010; Lyons & Ghetti, 2011; Roebers et al., 2004) or in adult studies with large samples (Rouault, Seow, et al., 2018). However, for some behaviours, sex differences may be heightened in adolescence (e.g. sex differences in risk‐taking behaviours are smaller for adults than adolescents; Byrnes et al., 1999). Therefore, a sex‐specific difference in confidence may be a unique feature of adolescence, but further investigations into sex differences across development are needed.

In our task, we used a neutral ‘space advisor’ without providing detailed information about their identity (age, gender etc.). We chose a neutral space advisor to eliminate any biases in advice taking that might arise from providing details about the identity of the advisor. An important next step would be to contrast types of advice/advisors that are known to moderate the social influence of an agent (e.g. Hertz & Wiese, 2016, 2018; Lourenco et al., 2015; Toelch & Dolan, 2015). For example, the current study did not distinguish between normative vs. informational influence, social vs. non‐social or peer vs. adult advisors. Adolescents appear to be influenced by peers more than non‐peers (e.g. adults or computers) in risk‐taking, moderating their behaviour simply in the presence of peers (Braams et al., 2019; Chein et al., 2011; Gardner & Steinberg, 2005; Reiter et al., 2019). Less is known about the effects of advice giving on adolescent behaviour. It may be the case, for example, that adolescents are less influenced by peers compared with adults when advice is informational (e.g. as in Lourenco et al., 2015) rather than normative (as in risk‐taking studies). Thus, it would be interesting to investigate whether enhanced metacognitive insight in adolescence is equally as protective against the influence of false advice in different social contexts.

Given the behaviour of participants in the current experiment, it may be that adolescents perceived the advisor as helpful but not all‐knowing whereas the children may have interpreted advice as highly reliable. Therefore, it may be interesting to explicitly assess the participants’ beliefs about the identity of the advisor and the validity of their advice, to investigate developmental changes in beliefs about others and whether this impacts on advice taking behaviours.

Confidence‐based measures, such as metacognitive bias and metacognitive efficiency, have been found to be associated with self‐esteem and psychiatric symptom dimensions such as anxious‐depression, compulsivity and intrusive thoughts (Moses‐Payne et al., 2019; Rouault, Seow, et al., 2018). Furthermore, post‐decision evidence integration, a similar process to advice taking, has been shown to be associated with depressive symptoms (Moses‐Payne et al., 2019). It would thus be particularly interesting to map the individual developmental trajectories of metacognition and advice taking behaviour in longitudinal studies. Since adolescence is a period of heightened risk for the onset of mental health conditions (Kessler et al., 2005) it would be interesting to investigate how trajectories of metacognitive development are associated with mental health symptom onset. Longitudinal studies could also help to overcome some of the caveats that arise in mediation analysis of cross‐sectional data (Lindenberger et al., 2011) and replication of the results in an independent sample is important, considering that often very large samples are needed to detect mediation effects in psychology (Fritz & MacKinnon, 2007; Schoemann et al., 2017).

The transition from childhood to adolescence is associated with major physical and psychological changes (Blakemore et al., 2010). Adolescents may be particularly driven to seek independence and gain more responsibility, and may spend more time thinking about their sense of self (Sebastian et al., 2008). In this study, we have shown that the ability to accurately introspect about our own behaviour improves from childhood to adolescence. Furthermore, we showed that adolescents harness this emerging ability to know when to take advice from others, and thus are able to take on helpful advice but ignore misleading advice. As Socrates implied, teenagers can sometimes appear ignorant towards others’ advice. However, what we have shown here, is that teenagers may actually be making quite sensible decisions to ignore the advice of others when they know that they are correct. Metacognition may thus be a driving force, supporting adolescent decision‐making and the transition towards full independence.

## CONFLICT OF INTEREST

The authors declare no competing financial interests.

## Supporting information

Supplementary Material

## Data Availability

Data is available at https://osf.io/h26fq/.
