# Supplementary material for: I know better! Emerging metacognition allows adolescents to ignore false advice
Source: Dev Sci. 2021 Mar 8;24(5):e13101. doi: 10.1111/desc.13101 (PMC8612133; doi:10.1111/desc.13101)
Supplement: Supplementary file 1 — Supplementary Material [file DESC-24-e13101-s001.docx]

**Supplementary – Adolescent advice-taking**

**Staircase**

We implemented a two-down one-up staircase procedure with equal step-sizes for steps up and steps down (Cornsweet, 1962; Fleming, Weil, Nagy, Dolan, & Rees, 2010; García-Pérez, 1998; Levitt, 1971). There was a starting point difference of 30 aliens between the colours (i.e. 49 colour A, 19 colour B) with a starting step size of ± 8 aliens which was halved after 10 reversals, halved again after 30 reversals, again after 50 and so on. The staircase was initiated during the 30 practice trials to minimize burn-in period and continued working throughout the task. The staircase was successful in matching the performance of our participants (between-group difference: *F*(2,104) = 0.36, η_p_^2^ = .01, *p* = .700) and could also account for any differences between groups in speed vs accuracy trade-offs.

**Signal detection performance measures – d’**

There were no differences in d’ (metric of performance) across the age groups (*F*(2,104) = .05, *p* = .947).


*Figure S1*: d’ (signal detection theory derived measure of performance) was matched across participants in each age group. Error bars show 95% confidence intervals.

**Evidence difference**

Younger participants required a greater evidence difference (number of alien colour A – number of alien colour B, excluding practice trials) in order to detect the more plentiful alien at the same performance level as the older participants (*F*(2) = 3.11, *p* = .049; Winning model linear: β = -.96, *p* = .013).


*Figure S2*: Mean evidence difference (number of alien A minus number of alien B). The difference needed to perform at the same accuracy decreased linearly across age. Error bars show 95% confidence intervals.

**Calculating metacognitive sensitivity and efficiency**

Metacognitive sensitivity (meta-d’) and efficiency (meta-d’/d’) are model-based measured developed by Maniscalco & Lau, (2012, 2014). Meta-d’ is based in signal detection theory, where type II (confidence) behavior is characterized by a 2 x 2 confidence-accuracy table and metacognitive sensitivity is essentially the difference between type II ‘hits’ (high confidence correct trials) and type II ‘false alarms’ (high confidence incorrect trials). Since these trials will not be normally distributed we use a non-parametric analysis (receiver operating characteristics – ROC) to assess metacognitive ability. ROC curves plot the proportion of high confidence trials when the participant is correct (hit rate) against the proportion of high confidence trials when the participant is incorrect (false alarm rate). To obtain the curve, confidence ratings are split by different criterion (e.g. low confidence = 1, high confidence = 2-4, then low confidence = 1-2, high confidence = 3-4 and so on), for each split hit and false alarm rate are calculated and plotted to obtain the type II ROC curve. The area under the curve (AUROC) can be used as a measure of metacognitive sensitivity and the higher the area under the curve the higher participants’ sensitivity.

The issue with simple AUROC analysis is that it is affected by type 1 performance (perceptual performance). This is where model-based measures come into play, as they exploit the fact that type II performance is constrained by type I performance i.e. if a participant performs at chance on the perceptual decision it is impossible for them to have any metacognitive insight into their performance (Galvin, Podd, Drga, & Whitmore, 2003). Therefore, we can place strong constraints on the measure of metacognitive sensitivity. Meta-d’ is then given in units of type I d’, so it can be thought of as the sensory evidence (both internal and external) available for metacognition in signal-to-noise ratio units in the same way that type I d’ is the sensory evidence available for decisions in signal-to-noise ratio units. Because of this, we can directly compare meta-d’ and d’. If they are equal, then the participant is an ideal observer; if meta-d’ is less than d’ then the participant is suboptimal. Metacognitive efficiency is therefore defined as meta-d’/d’. We used Matlab code available online to compute metacognitive efficiency (<http://www.columbia.edu/~bsm2105/type2sdt/>).

For further information on the development and calculation of metacognitive sensitivity and efficiency we point the readers to Maniscalco & Lau (2012, 2014) and Fleming & Lau (2014)

**Performance difference pre (all trials excluding practice) - post advice**

While older groups remained at the same performance level after receiving advice (as expected if follow advice sensibly because the advice has the same accuracy level as the participants), the youngest participants performed worse following advice (*F*(2) = 2.81, *p* = .065; Winning model emergent: β = .02, *p* = .025). The youngest participants performed worse after advice (single-sample t-test *t*(29) = -3.10, *p* = .004) whereas the other groups performed at the same level (12-13 yo: *t*(40) = -1.16, *p* = .252; 16-17 yo: *t*(35) = -1.26, *p* = .215).

*Figure S3*: Percentage difference in performance from pre-advice to post-advice. Error bars show 95% confidence intervals.

**Age curves**


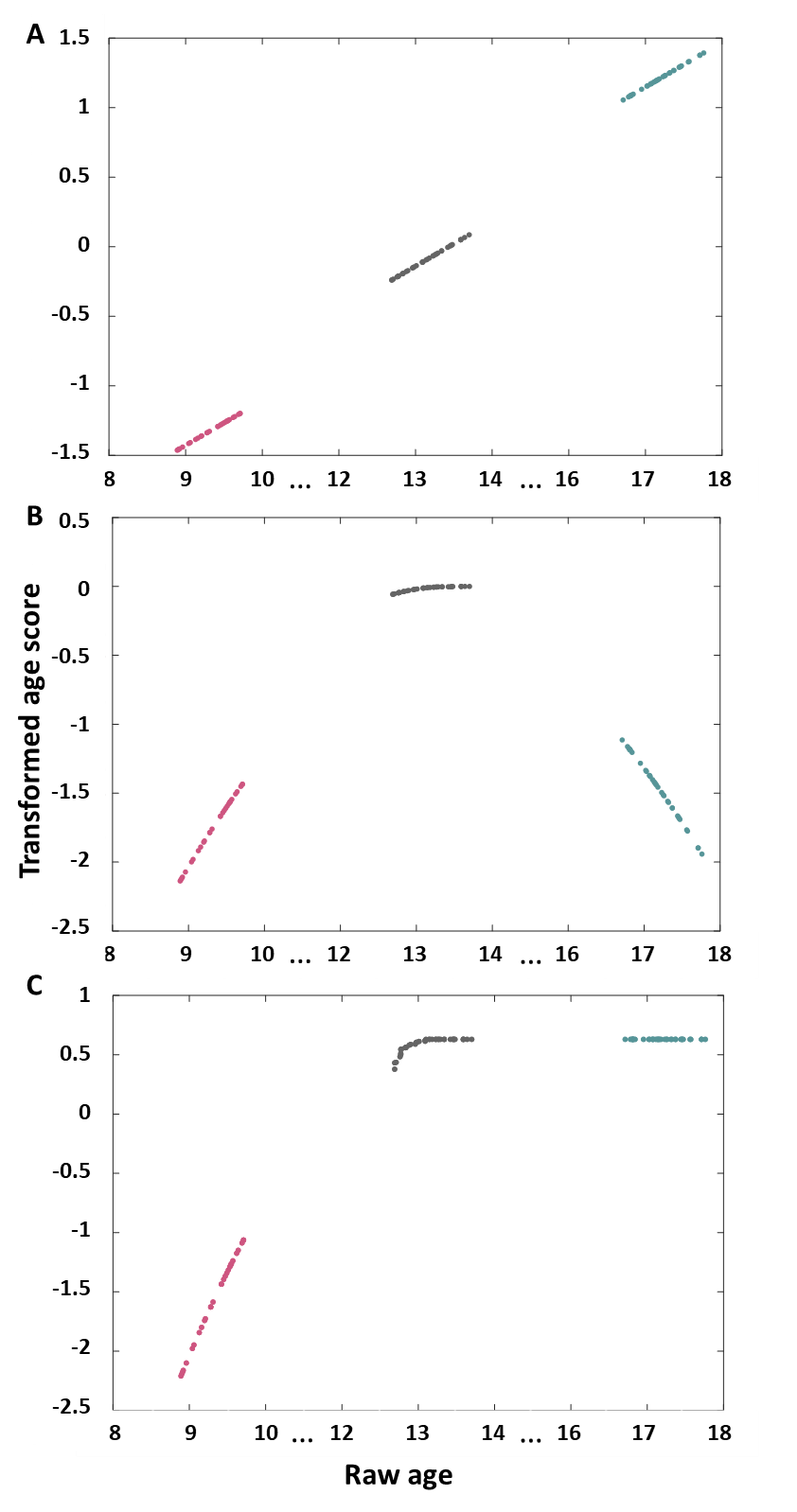


*Figure S4*: Standardized age scores, transformed for model comparison analysis. A) Linear age score. Calculated using the standardized raw age scores. B) Quadratic age score. Calculated using the standardized raw age scores squared. C) Emergent age score. Calculated using the standardized raw age scores squared and then changing all values above age twelve as the same standardized value.

**Influence of feedback in practice trials**

The first 30 trials of the space explorer task were included as practice trials. Unlike the experimental trials, participants were given feedback on their perceptual decision and did not have to give confidence ratings or decide whether to take advice. This was to ensure faster convergence of the staircase procedure. All participants started at the same evidence difference (number of aliens in colour A vs colour B) and as the staircase converged, younger participants required a greater evidence difference to remain at the same performance as older participants. Therefore, the younger participants received greater numbers of trials in which they were incorrect (and received ‘incorrect’ feedback) in the practice trials (linear age: β = -.24, p = .013; 8-9yo M ± SD = 8.33 ± .84; 12-13yo M ± SD = 8.02 ± .65; 16-17yo M ± SD = 7.89 ± .67; Total practice trials = 30). We therefore analysed whether this increased negative feedback in the practice trials had an influence on confidence-related measures in the experimental trials. We did not find any evidence of an influence of the amount of negative feedback in practice on i) overall confidence (feedback: β = .35, p = .725; age and sex remain significant) or ii) metacognitive efficiency (feedback: β = -.0004, p = .993; age remains significant).

**Use of the confidence scale across age groups**

Figure S5: Participants’ confidence standard deviation (SD) across age groups. Participants in different age groups seemed to use the confidence scale in a similar way. Participants from different age group showed a similar SD in their confidence ratings (*F*(2) = 1.63, *p* = .202).


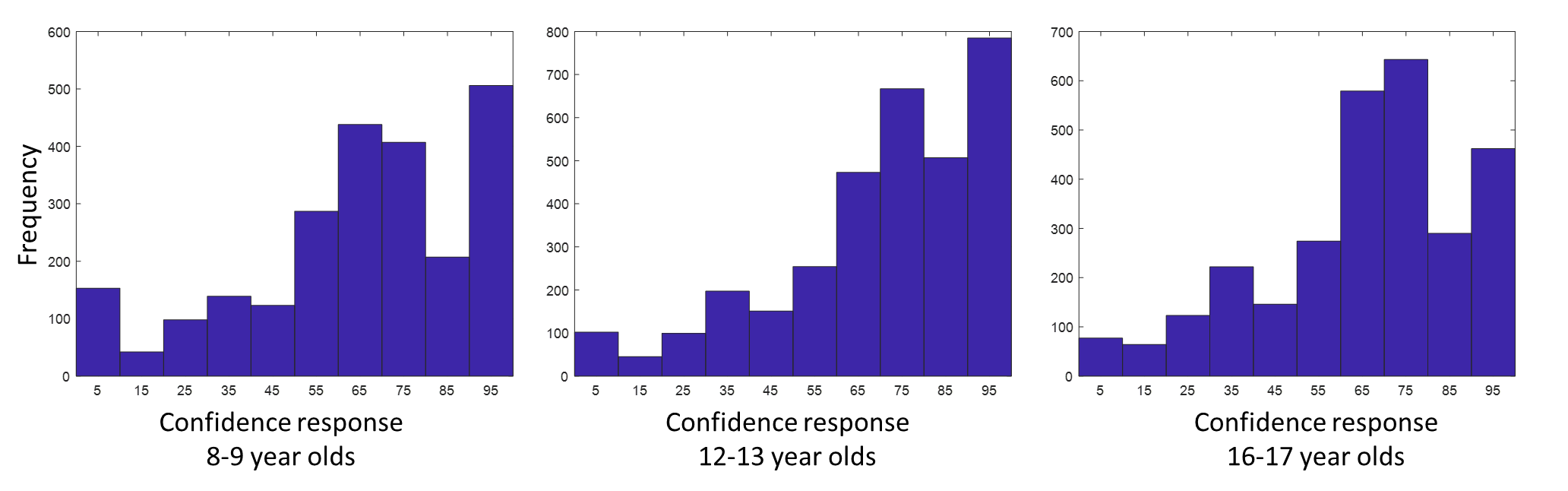


Figure S6: Participants confidence distributions across age groups. Participants confidence responses were well distributed across the scale, showing a similar pattern of responses across age groups.

**BIC tables and full results**

*Table S1*: Bayesian Information Criteria, Beta estimates and p-values for analyses reported in the main transcript. Values in bold represent the winning model. Meta bias – metacognitive bias, overall mean confidence. Meta-d’/d’ – metacognitive efficiency. Advice-taking – propensity to follow advice. Resist false ad – resistance to false advice.

| BIC | Linear | Quadratic | Emergent | L + Q | L + E | E + Q | L + Q + E |
| --- | --- | --- | --- | --- | --- | --- | --- |
| Meta bias | 311.98 | **307.09** | 310.78 | 311.77 | 313.48 | 311.70 | 313.26 |
| Meta-d’/d’ | 298.87 | 301.78 | **298.12** | 301.99 | 302.57 | 302.76 | 305.38 |
| Advice-taking | 305.16 | 310.11 | **303.78** | 308.24 | 308.24 | 308.29 | 312.89 |
| Resist false ad | 309.92 | 308.61 | **307.39** | 311.45 | 311.71 | 311.55 | 316.09 |
| Estimates (β) | Linear | Quadratic | Emergent | L + Q | L + E | E + Q | L + Q + E |
| Meta bias | .01 | **.27** | .11 | L: .0001 Q: .27 | L: -.24  E: .30 | E: -.03 Q: .29 | L: 1.11  Q: 1.23  E: -1.40 |
| Meta-d’/d’ | .21 | .16 | **.22** | L: .20  Q: .15 | L: .07  Q: .16 | E: .23  Q: -.01 | L: .93 Q: .77  E: -.90 |
| Advice-taking | -.25 | -.17 | **-.27** | L: -.24  Q: -.15 | L: -.08  E: -.21 | E: -.30 Q: .06 | L: -.16  Q: -.08  E: -.10 |
| Resist false ad | .14 | .22 | **.21** | L: .13  Q: .21 | L: -.10  E: .29 | E: .16  Q: .11 | L: .23  Q: .30  E: -.13 |
| p-values | Linear | Quadratic | Emergent | L + Q | L + E | E + Q | L + Q + E |
| Meta bias | .898 | **.029** | .276 | L: .999  Q: .030 | L: .168  E: .080 | E: .796 Q: .057 | L: .084  Q: .030  E: .081 |
| Meta-d’/d’ | .035 | .206 | **.023** | L: .038  Q: .226 | L: .662  Q: .340 | E: .060  Q: .936 | L: .164  Q: .185  E: .272 |
| Advice-taking | .010 | .175 | **.005** | L: .012  Q: .215 | L: .653  E: .215 | E: .012  Q: .692 | L: .799  Q: .887  E: .901 |
| Resist false ad | .155 | .069 | **.034** | L: .183  Q: .081 | L: .563  E: .095 | E: .196  Q: .484 | L: .720  Q: .596  E: .871 |

**Metacognitive efficiency (bounded analysis)**

We analysed metacognitive efficiency using meta-d’/d’, which uses maximum likelihood estimation and a signal detection theory approach to get a bias-free measure of how well participants’ confidence ratings track their performance. Metacognitive sensitivity (meta-d’) is theoretically bound at zero but, in practice, negative values can occur (when fit using an unbounded maximum likelihood estimation procedure). As this estimation error applies to all values, we did not adjust negative values in the main analysis (in the manuscript). However, for completeness we provide this analysis where negative meta-d’ values are set to zero.

We repeated our linear regression analysis, assessing the effect of age on metacognitive efficiency (meta-d’/d’) with sex as a covariate. As in the unbounded analysis, there was a significant effect of age (adolescent-emergent: β = .20, *SE* = .10, *p* = .044).

Mediation showed the same pattern of results as in the unbounded analysis (*a*: mean β = .20, *SE* = .08, *z* = 2.50, *p* = .012; *b*: mean β = .29, *SE* = .11, *z* = 2.42, *p* = .016; *c’*: mean β = .15, *SE* = .09, *z* = 1.71, *p* = .088; *c*: mean β = .21, *SE* = .10, *z* = 2.24, *p* = .025; *ab*: mean β = .06, *SE* = .03, *z* = 2.54, *p* = .011). Again, showing that metacognitive efficiency mediated the effect of age on resistance to false advice.

**Metacognitive efficiency**

*Figure S7*: Metacognitive efficiency with values bounded at the lower end at zero. Error bars represent 95% confidence intervals. There was a significant effect of adolescent-emergent age on bounded metacognitive efficiency (meta-d’/d’).

**Post-advice confidence**

There was no difference in post-advice metacognitive bias (recoded to reflect confidence in initial choice) across age groups (*F*(2,104) = 2.19, *p* = .117) or across sex (*t*(105) = 1.24, *p* = .220).

We calculated the difference between metacognitive efficiency post-advice minus pre-advice and found no evidence of age-related change in metacognitive efficiency improvement (*F*(2,99) = .65, *p* = .522).

There were also no significant age-related differences in the extent to which participants increased their confidence when the advisor agreed with them (*F*(2,104) = 1.24, *p* = .293) or decreased their confidence (in their initial choice) when the advisor disagreed (*F*(2,104) = 1.51, *p* = .226).

**References**

Cornsweet, T. N. (1962). The staircase-method in psychophysics. *The American Journal of Psychology*. https://doi.org/10.2307/1419876

Fleming, S. M., & Lau, H. C. (2014). How to measure metacognition. *Frontiers in Human Neuroscience*, *8*. https://doi.org/10.3389/fnhum.2014.00443

Fleming, S. M., Weil, R. S., Nagy, Z., Dolan, R. J., & Rees, G. (2010). Relating introspective accuracy to individual differences in brain structure. *Science*. https://doi.org/10.1126/science.1191883

Galvin, S. J., Podd, J. V., Drga, V., & Whitmore, J. (2003). Type 2 tasks in the theory of signal detectability: Discrimination between correct and incorrect decisions. *Psychonomic Bulletin and Review*. https://doi.org/10.3758/BF03196546

García-Pérez, M. A. (1998). Forced-choice staircases with fixed step sizes: Asymptotic and small-sample properties. *Vision Research*. https://doi.org/10.1016/S0042-6989(97)00340-4

Levitt, H. (1971). Transformed Up‐Down Methods in Psychoacoustics. *The Journal of the Acoustical Society of America*. https://doi.org/10.1121/1.1912375

Maniscalco, B., & Lau, H. (2012). A signal detection theoretic approach for estimating metacognitive sensitivity from confidence ratings. *Consciousness and Cognition*. https://doi.org/10.1016/j.concog.2011.09.021

Maniscalco, B., & Lau, H. (2014). Signal detection theory analysis of type 1 and type 2 data: Meta-d′, response-specific meta-d′, and the unequal variance SDT model. In *The Cognitive Neuroscience of Metacognition* (Vol. 9783642451, pp. 25–66). https://doi.org/10.1007/978-3-642-45190-4_3
